# Single-shot deflectometry for dynamic 3D surface profile measurement by modified spatial-carrier frequency phase-shifting method

**DOI:** 10.1038/s41598-019-39514-6

**Published:** 2019-02-28

**Authors:** Manh The Nguyen, Young-Sik Ghim, Hyug-Gyo Rhee

**Affiliations:** 1 Space Optics Team, Advanced Instrumentation Institute, Korea Research Institute of Standards and Science (KRISS), Science Town, Daejeon, 34113 South Korea; 2 Department of Science of Measurement, University of Science and Technology(UST), Science Town, Daejeon, 34113 South Korea

## Abstract

We propose a new concept of single-shot deflectometry for real-time measurement of three-dimensional surface profile using a single composite pattern. To retrieve an accurate phase from one-frame composite pattern, we adapt the Fourier Transform (FT) method and the spatial carrier-frequency phase-shifting (SCPS) technique to our proposed deflectometry. Based on Lissajous figure and ellipse fitting method, we also correct the phase extraction error in SCPS technique by reducing the effect of background and modulation variations. The proposed technique is verified by comparing our measurement results with phase-shifting deflectometry, and the maximum difference between two measurement results is less than 30 nm rms. We also test the robustness to vibration and the measurement capability for dynamic object.

## Introduction

Three-dimensional (3D) shape measurements of freeform surfaces using non-contact optical methods have become attractive solutions for the effective quality control of products to a variety of industrial applications such as car body panels, smartphone cover glass, and smart glasses. Among existing 3D shape measurement technologies, deflectometry-based surface metrology technique is the most promising candidate for 3D surface measurement of free-form shape providing distinct advantages of large dynamic range, non-contact operation, full-field, automatic data-processing and high precision^1,2^. In general, deflectometric method utilizes phase-shifting technique^3–5^ (PS) for reconstruction of the shape of surfaces by obtaining at least six fringe patterns (three in the horizontal and three in the vertical direction). This phase-shifting deflectometry (PSD) is quite time consuming and sometime not so suitable for industrial applications that are essential for measurements of large test samples and high speed. Moreover, many measurement applications in practice are performed in harsh environmental conditions such as vibration and air turbulence. Therefore, much research has been done to achieve high speed 3D shape in real-time or robustness to environmental noises, and single-shot approaches using just one image to reconstruct the surface shape of object will be one of suitable solutions for industrial metrology. Most of these single-shot techniques take the advantage of Fourier transform technique^6^. For example, J. L. Flores *et al*. proposed one-frame deflectometry technique by addition of orthogonal fringe patterns^7^. L. Huang *et al*. presented a dynamic three-dimensional sensing for specular surface with monoscopic fringe reflectometry^8^. These methods are simple and fast, but they have disadvantages of sensitive to frequency filtering process which make uncertainty in surface profile. It means that it will give the different results of surface profile according to each filter shape and size^9^. This is undesirable in measurement technology. Moreover, frequency leakage and boundary effect which come from Gibb effect and global operation are inherent properties of Fourier Transform (FT) method, which will cause edge error^10–12^. This kind of error could be mitigated by some local Fourier technique such as windowed Fourier transform^13^ or wavelet-transform^14^, but the computational complexity is relatively high. Hilbert transform (HT) is also used to measure the 3D surface profile from a single fringe pattern^15^ due to simple calculation and short calculation time. However, this method has two disadvantages: it may cause errors at the edge like FT method if the cycle number of the signal is not complete and the phase generated by HT method will have additional phase wrapping problem if the fringe pattern is closed^16^. Another one-shot technique is the instantaneous phase-shifting deflectometry proposed by I. Trumper *et al*.^9^. This method uses similar fringe pattern as previous approaches, but the main difference is color modulation and they utilize conventional three-steps phase-shifting techniques for phase retrieval instead of Fourier transform. Therefore, it can avoid errors come from Fourier-based techniques. But, it also has its own disadvantages, such as color cross-talk between each color channel and color absorption of surface materials which restricts its use. There are also other types of single shot techniques^17,18^ and most of them utilize FT method which will be suffered from errors we mentioned above.

Until now, single-shot deflectometry has been demonstrated by two ways of FT and method using a gray scale camera^7,8,17,18^ and multiplexing phase-shifting method using a color camera^9^. The FT method can be free from the cross-talk error between color channels, but it will have a considerable edge error. Meanwhile, the phase-shifting method using a color camera can avoid the edge error but it will suffer from cross-talk errors. In order to overcome these technical limitations of conventional methods, an improved version of single-shot deflectometry is needed. In this paper, we introduce a new concept of single-shot deflectometry which not only avoids all of the disadvantages mentioned above but also gives as equivalent or superior performance as phase-shifting algorithm. Moreover, our proposed method can provide three-dimensional surface profile measurement for dynamic object in real time.

## Method

### Basic principle of deflectometry

**Figure 1:**
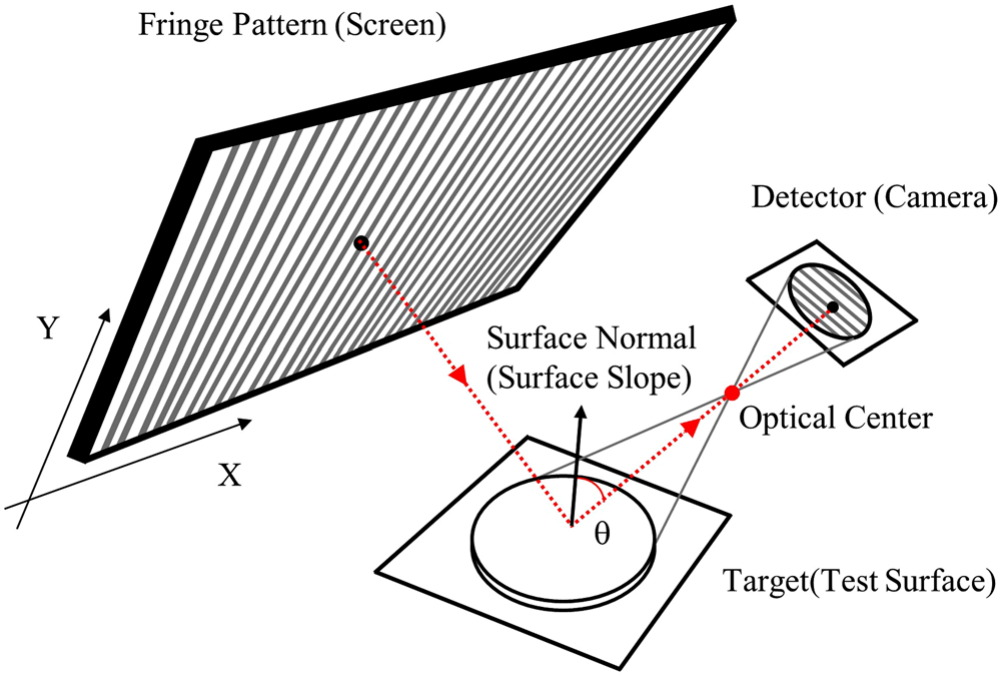
Schematic diagram of deflectometry system.

Deflectometry is reflective surface measurement technique which measures the slope data directly with very large dynamic range and requires simple hardware. Basic deflectometry system consists of a screen to display pattern, and a camera to capture the distorted images of test surface, which is illuminated by screen. In order to calculate the slope of test surface, it is essential to know which screen pixel reflected from the test surface corresponds to which camera pixel. The mapping between camera pixel and screen pixel gives information about the surface slope of target. There are many techniques to create this kind of mapping and they can be divided into two main categories as direct mapping and indirect mapping. Direct mapping method such as line scanning can find this mapping by intensity level of captured images from camera directly^1^. And, indirect method such as phase-shifting or Fourier transform uses the phase information to retrieve this mapping. Through these mapping procedures, the surface slope in two orthogonal directions can be calculated. Figure 1 shows the general schematic of deflectometry system.

In order to measure the surface profile using deflectometry, there are several steps as follows

1. Generate fringe patterns by computer and project these patterns to test surface by screen.
2. Capture reflected patterns by camera.
3. Position mapping between screen and camera caused by target using direct or indirect method
4. Calculate two orthogonal slopes based on position mapping.
5. Reconstruct 3D surface shape.

In generally, most of deflectometry methods are similar in all steps. The difference here is in step (1) and step (3) where they implemented various ways to generate projecting patterns and corresponding technique to make position mapping. Our proposed method also focuses on these two steps and other steps are still kept the same as other deflectometry methods.

### Single-shot deflectometry for dynamic 3D shape measurement

In our proposed method, we still take the advantage of using composite pattern by the addition of orthogonal fringe patterns which is similar to the previous method proposed by I. Trumper *et al*.^9^. The difference here is that we use gray scale pattern instead of color composite pattern and this will increase the scalability of applications. The main focus here is on how to find the mapping between screen and camera pixels from a single-shot gray scale image. We divided our proposed method into three smaller steps as follows.

1. Decompose captured composite pattern from camera into two single patterns in x- and y-directions.
2. Single patterns in x- and y-directions are then normalized by use of Lissajous figure and ellipse fitting to reduce the effect of background and modulation variations and create sub-patterns from them.
3. Retrieve the phase maps of x- and y-directional patterns using well-known least-squares iteration algorithm.

**Figure 2:**
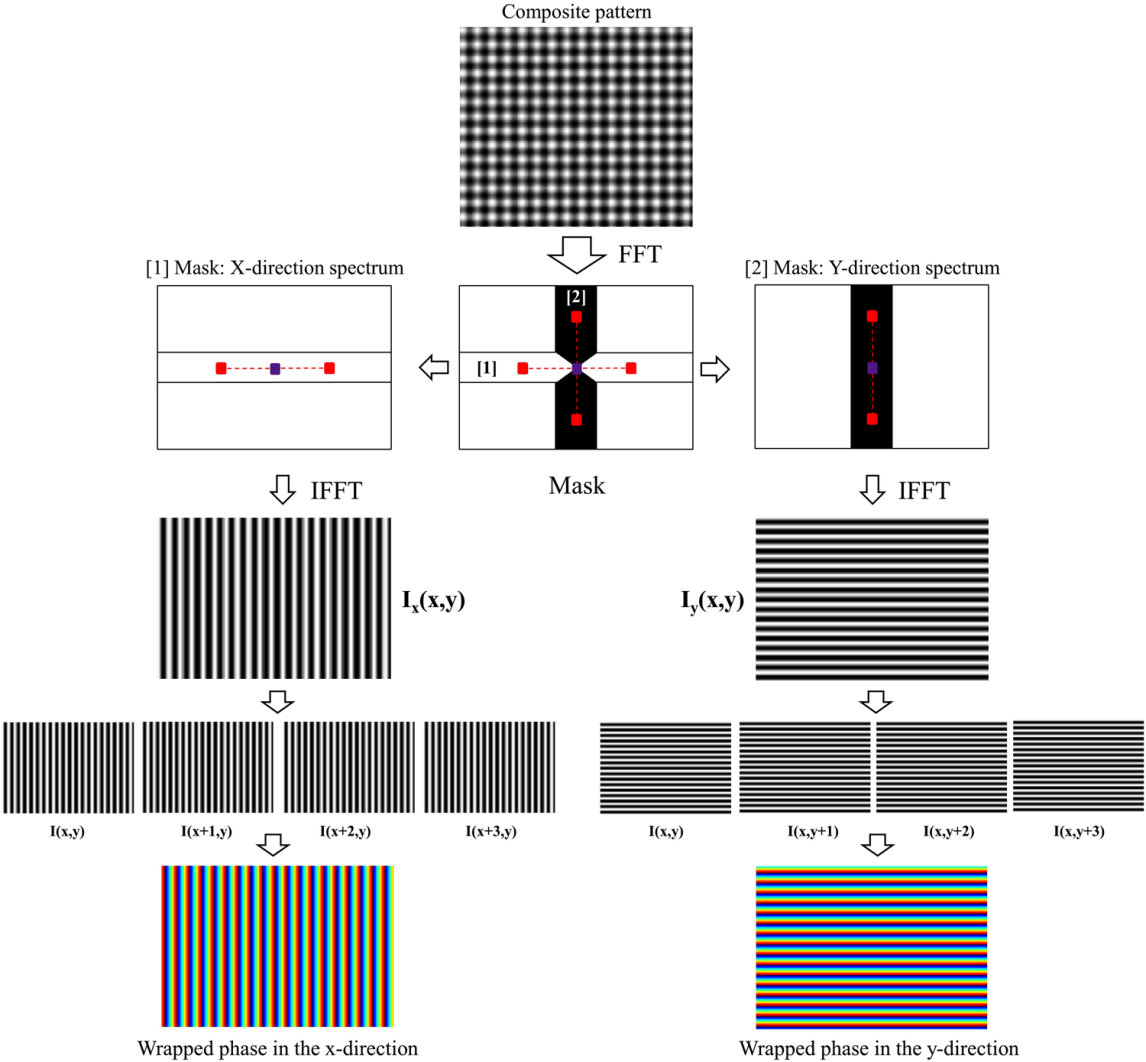
Overview of our proposed one-shot deflectometry technique. Note that this is not overall data processing of deflectometry. This is just the main contribution steps, remained processing steps are similar to most of deflectometry system. Therefore, they are omitted for clarity.

The main contribution of this paper locates at the step 2 and 3, where we proposed to apply spatial-carrier phase-shifting (SCPS) method for the phase retrieval of two fringe patterns after the decomposition of a composite pattern. We do not only propose the use of this method but also modify the conventional SCPS to make it suitable for our deflectometry system. As far as we know, this is the first time SCPS method is modified and applied into deflectometry to achieve dynamic 3D shape measurement. Detailed explanation of each step will be described throughout this paper. Figure 2 summarizes these steps for demonstration.

For convenience, we define the two orthogonal directions of the fringe to be in x and y directions. Composite pattern can be expressed as

1 $${I}_{composite}=(\frac{G}{2})(2+\,\cos ({w}_{x}{x}_{s})+\,\cos ({w}_{y}{y}_{s}))$$

where G is a constant that presents the amplitude value to obtain the maximum gray level range (G = 255 for eight bit images); w_x_, w_y_ are carrier frequencies in horizontal and vertical directions; (x_s_, y_s_) are x and y pixel coordinate of screen. Carrier frequencies should be chosen as high as possible in order to obtain more accurate phase which will be explained in the next part. Instead of projecting several phase-shifting patterns in x and y directions separately, we only need to project this single composite pattern one time. Captured fringe pattern from camera will be used to retrieve phase using the method described in the next part.

### Phase retrieval from single composite pattern with modified spatial-carrier phase-shifting method

**Figure 3:**
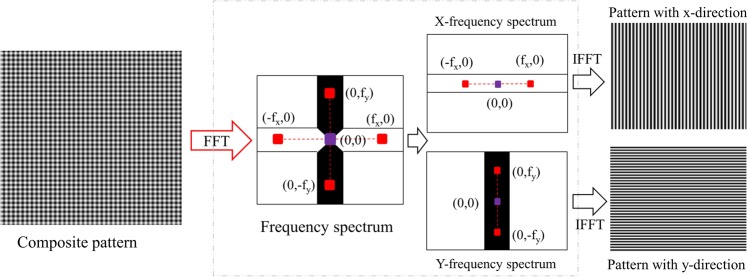
Procedure showing how to decompose composite pattern into two single fringe patterns using Fourier transform. f_x_ and f_y_ are carrier frequencies in the frequency spectrum. f_x_ and f_y_ are determined by finding the position of maximal spectra in the frequency domain while excluding the zero-frequency peak.

Captured composite pattern will be decomposed into two separate patterns in x and y directions by the use of Fourier transform. With high carrier-frequencies, we can easily observe four peaks marked as red in Fig. 3 beside the center frequency (zero-order spectrum) in frequency spectrum after taking Fourier transform on composite pattern. These spectrums are first-order spectrum and they are conjugated in x- and y- directions, respectively. Two rectangular frequency masks called X and Y mask are used to select the information of x- and y-directional fringe patterns. The width of these masks is chosen carefully to avoid overlapping with other frequency spectrums. The zero-order spectrum is used in both filter masks to preserve the mean intensity value. By applying these masks to original frequency spectrum, we can separates out two spectrum group (X- and Y-frequency spectrums). Each group contains zero-order spectrum, first-order and its conjugate to preserve the details of the fringe pattern contained in each frequency direction. We then apply an inverse Fourier transform to each spectrum group to reconstruct x- and y-directional fringe patterns that make up the captured composite pattern.

Since the carrier frequency denoted as w_x_ and w_y_ in Eq. (1) is used to generate composite pattern, we can retrieve the phase distribution of distorted pattern by spatial carrier-frequency methods^19–21^. Theoretically, using higher carrier frequencies results in better phase retrieval accuracy. However, the maximum carrier frequency will be restricted due to limited screen and camera resolution. Therefore, we need to adjust these frequencies until obtaining proper fringe pattern in real experiment.

**Figure 4:**
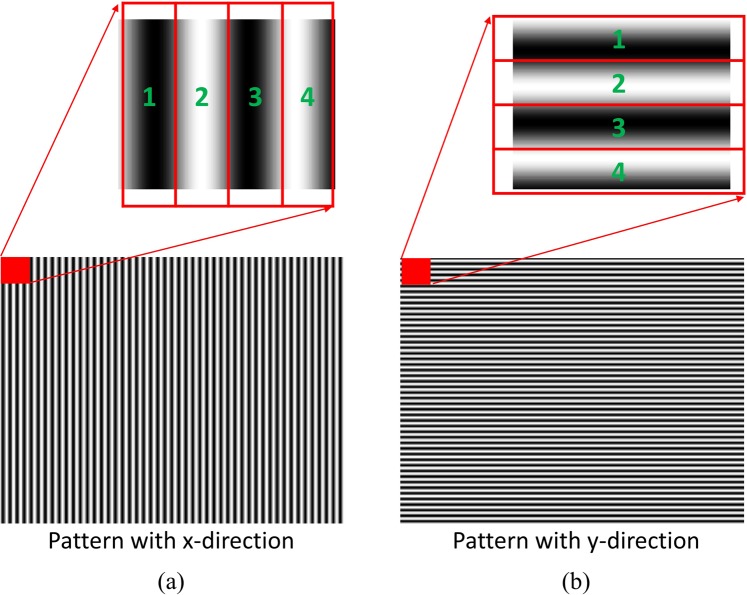
Method for constructing sub-fringe pattern in SCPS: (**a**) our modified SCPS method for x-directional fringe pattern and (**b**) our modified method for y-directional fringe pattern. The red square present group of four consecutive pixels starting from single pixel position (x, y) in the original pattern. Sub-fringe patterns are constructed from consecutive pixels numbered from 1 to 4.

In our proposed method, we modified the conventional spatial carrier-frequency method to make it suitable to deflectometry system but the basic principle is almost similar. First, we consider the x-directional fringe pattern obtained from composite pattern for demonstration. The intensity distribution of this pattern can be expressed as

2 $${I}_{x}(x,y)=A(x,y)+B(x,y)\cos (\phi (x,y)+{k}_{x}x)$$

where A(x, y), B(x, y), and φ(x, y) represent the background intensity, modulation amplitude, and phase distribution of the test sample, respectively. k_x_ is spatial carrier frequency along x-direction. Since there is only k_x_ frequency presented, intensity will change along x-direction only. Here, we can create four sub-patterns I_1x_(x, y) = I_x_(x, y), I_2x_(x, y) = I_x_(x + 1, y), I_3x_(x, y) = I_x_(x + 2, y), and I_4x_(x, y) = I_x_(x + 3, y). Figure 4 shows how to create four sub-fringe pattern using our modified SCPS method.

**Figure 5:**
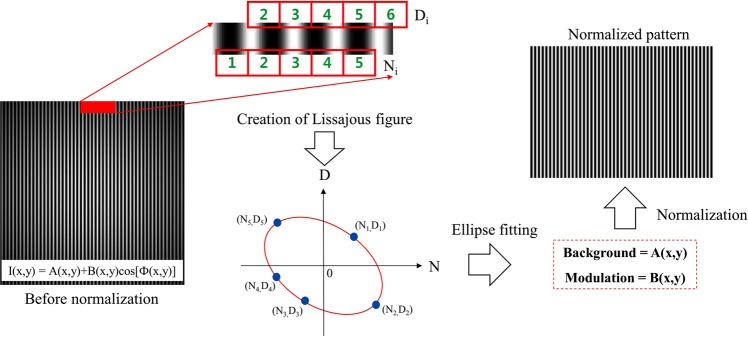
Construction of Lissajous figure and demodulation of background and modulation intensites by normalizing fringe pattern.

From discussion above, four-frame phase-shifting sub-patterns with size (N_row_ − n_o_) × (N_col_ − m_o_) can be constructed; where N_row_ and N_col_ are number of row and column of x-directional fringe pattern, n_o_ and m_o_ are the number to define the desired size of sub-patterns (minimum value of n_o_ and m_o_ is 3 for constructing four sub-patterns)

3 $$\begin{array}{lllll}{I}_{1x}(x,y) & = & {I}_{x}(x,y) & = & {A}_{1}(x,y)+{B}_{1}(x,y)\cos ({\rm{\Phi }}(x,y)+{\delta }_{1}(x,y))\\ {I}_{2x}(x,y) & = & {I}_{x}(x+1,y) & = & {A}_{2}(x,y)+{B}_{2}(x,y)\cos ({\rm{\Phi }}(x,y)+{\delta }_{2}(x,y))\\ {I}_{3x}(x,y) & = & {I}_{x}(x+2,y) & = & {A}_{3}(x,y)+{B}_{3}(x,y)\cos ({\rm{\Phi }}(x,y)+{\delta }_{3}(x,y))\\ {I}_{4x}(x,y) & = & {I}_{x}(x+3,y) & = & {A}_{4}(x,y)+{B}_{4}(x,y)\cos ({\rm{\Phi }}(x,y)+{\delta }_{4}(x,y))\end{array}$$

where A_n_(x, y), B_n_(x, y) and *δ*_*n*_(x, y) (n = 1, 2, 3, 4) are background, modulation and phase shift of each sub-pattern. Typically, A_n_(x, y), B_n_(x, y) are assumed to slowly vary in the pattern. Then, we can assume that the variation and background of fringe pattern at four-consecutive adjacent pixels are similar or same: A(x, y) ≈ A_1_(x, y) ≈ A_2_(x, y) ≈ A_3_(x, y) ≈ A_4_(x, y) and B(x, y) ≈ B_1_(x, y) ≈ B_2_(x, y) ≈ B_3_(x, y) ≈ B_4_(x, y). In another word, the intensity of four adjacent pixels in the pattern changed only with carrier-frequency. Then, we can consider that four sub-patterns in Eq. (3) are four phase-shifted fringe patterns which are similar to phase-shifting technique. The amount of phase shift *δ*_*n*_(x,y) in Eq. (3) is local phase shift. This is the actual phase shift value which varies from the global value or averaged phase shift based on actual profile of test surface. It is important to obtain these local phase shifts in order to retrieve the surface map accurately. Most of spatial carrier-frequency methods calculate the phase map under the assumption described above. However, deflectometry is full-field fringe projection technique, the measurement area is usually large. Therefore, the background and modulation intensity might change abruptly in some measurement scenarios. Moreover, the measurement environment is complex and not ensure the assumption above is maintained. Therefore, we need to make sure the variations of background and modulation of fringe pattern are slowly changed. This can be done by normalizing the fringe pattern before constructing four sub-patterns. We can demodulate the fringe pattern into its background and modulation separately by use of Lissajous figure and ellipse fitting^22^. As shown in Fig. 5, if we denote the intensity level at pixel [1 2 3 4 5] is N_i_ and at pixel [2 3 4 5 6] is D_i_ (i = 1, 2, 3, 4, 5) and plot N_i_ over D_i_, a Lissajous figure can be constructed. The minimum number of point is five required to construct an ellipse. We can increase this number to enhance the stability of ellipse construction robust to noise. The 2D mode of creating Lissajous figure can be used for relatively lower carrier frequencies and higher noise levels^23^. We only present 1D mode here for simplicity. By using least square fitting method, we can find the coefficients of the following conic equation expressed as^22,24^

4 $${C}_{1}{I}_{1}^{2}+2{C}_{2}{I}_{1}{I}_{2}+{C}_{3}{I}_{2}^{2}+2{C}_{4}{I}_{1}+2{C}_{5}I{}_{2}\,+\,{C}_{6}=0$$

where I_1_ and I_2_ equal N_i_ and D_i_ respectively; C_i_(i = 1, 2, 3, 4, 5, 6) is ellipse coefficient. Then, the background and modulation of fringe pattern can be calculated by

5 $$A(x,y)=\frac{{C}_{2}{C}_{5}-{C}_{3}{C}_{4}}{\alpha },B(x,y)=\frac{\sqrt{-{C}_{3}{\rm{\Delta }}}}{\alpha }$$

where *α* and *∆* are defined as

$$\alpha =|\begin{array}{cc}{C}_{1} & {C}_{2}\\ {C}_{2} & {C}_{3}\end{array}|,\,{\rm{\Delta }}=|\begin{array}{ccc}{C}_{1} & {C}_{2} & {C}_{4}\\ {C}_{2} & {C}_{3} & {C}_{5}\\ {C}_{4} & {C}_{5} & {C}_{6}\end{array}|$$

With obtained background and modulation intensities, we can normalize the fringe pattern using the following equation

6 $${\tilde{I}}_{x}(x,y)=\frac{{I}_{x}(x,y)-A(x,y)}{B(x,y)}$$

Once the fringe pattern is normalized, the assumption will be valid in most of measurement condition. Then, we can retrieve the phase map from these two single patterns by several spatial carrier frequency phase-shifting methods mentioned above. In order to achieve fast speed, high accuracy, and superior performance by compensating local phase shift and mitigating edge error, we will use the hybrid algorithm for phase retrieval from a single spatial carrier fringe pattern. The detailed explanation of this method was presented by Z. Dong and H. Cheng^19^. But, for completeness of this paper, we will briefly describe the main steps in this method. From a single normalized pattern, we can construct four phase-shifted sub-patterns and construct more sub-patterns from more consecutive and adjacent pixels of the original fringe pattern. In general, the noise tolerance of phase retrieval is better if more sub-patterns are constructed^21^. Since four sub-patterns are sufficient enough to obtain reliable and accurate phase map, we do not need to construct more sub-patterns to save processing time and computation resource. Four phase-shifted sub-pattern fringes are constructed by same modified SCPS and be written as

7 $${\mathop{I}\limits^{ \sim }}_{nx}(x,y)={\mathop{A}\limits^{ \sim }}_{n}(x,y)+{\mathop{B}\limits^{ \sim }}_{n}(x,y)\cos ({\rm{\Phi }}(x,y)+{\delta }_{n}(x,y))$$

where ${\mathop{A}\limits^{ \sim }}_{n}(x,y)$ and ${\tilde{B}}_{n}(x,y)$ are background and modulation of normalized fringe patterns. Ideally, ${\mathop{A}\limits^{ \sim }}_{n}(x,\,y)$ equals 0 and ${\tilde{B}}_{n}(x,\,y)$ equals 1 at every pixel on the normalized pattern. The value ${\delta }_{n}(x,\,y)$ denotes the phase shift of nth fringe pattern at pixel (x, y) with ${\delta }_{n}(x,y)\approx (n-1){w}_{x}$. The averaged phase shifts or the global phase shifts between each sub-pattern with the original x-directional pattern are 0, w_x_, 2w_x_, and 3w_x_, respectively. Then, FT method is used to retrieve the phases from each sub-fringe pattern^6^. Here, we denote these phases as *φ*_*1x*_  *(x, y), φ*_*2x*_  *(x, y), φ*_*3x*_  *(x, y), φ*_*4x*_  *(x, y)*. Note that these phases are obtained without shifting frequency spectrums to the center as previous Fourier-based methods, because we need to obtain the carrier frequency phases as well. These phases also contain the local phase shifts between each sub-fringe pattern. Then, the amount of phase shift between each pattern can be calculated as follows

8 $${\delta }_{n}(x,y)=unwrap[{\phi }_{nx}(x,y)]-unwrap[{\phi }_{1x}(x,y)].$$

where *unwrap* means phase unwrapping operator to resolve phase unambiguity such as Goldstein phase unwrapping algorithm^25^. Because the subtraction operation is used to determine the local phase shifts between selected pixels in each sub-fringe pattern, it can considerably mitigate the inherent edge error of FT method. That is because the four phase maps retrieved from four phase-shifted fringe patterns suffer from very similar edge errors with each other^19^. Moreover, local phase shifts are much smaller compare to global phase shift value. Therefore, the error come from FT method will be negligible in practice. With obtained local phase shift values, a least-squares algorithm can be used to retrieve the phase map. Since the background intensity and modulation amplitude do not have frame-to-frame variations, Eq. (7) can be expressed as:

9 $${\tilde{I}}_{nx}(x,y)=\mathop{A}\limits^{ \sim }(x,y)+V(x,y)\cos ({\delta }_{n}(x,y))+S(x,y)\sin ({\delta }_{n}(x,y))$$

where $V(x,y)=\tilde{B}(x,y)\cos ({\rm{\Phi }}(x,y))$, $S\,(x,y)=-\,\tilde{B}(x,y)\sin ({\rm{\Phi }}(x,y))$. When it is assuming that the measured value of ${\tilde{I}}_{nx}(x,y)$ is ${{\tilde{I}}^{e}}_{nx}(x,y)$, the error function of ${E}_{x}(x,y)$ can be defined by the least-squares method as follows

10 $${E}_{x}(x,y)=\sum _{n=1}^{N}{({\tilde{I}}_{nx}^{e}(x,y)-{\tilde{I}}_{nx}(x,y))}^{2}$$

where N denotes total number of patterns. From Eqs (9) and (10), there are three unknown values of $\mathop{A}\limits^{ \sim }(x,y)$, $V(x,y)$ and $S(x,y)$, which can be found by minimizing the error ${E}_{x}(x,y)$ with the following condition as

11 $$\frac{\partial {E}_{x}(x,y)}{\partial \tilde{A}(x,y)}=\frac{\partial {E}_{x}(x,y)}{\partial V(x,y)}=\frac{\partial {E}_{x}(x,y)}{\partial S(x,y)}=0$$

Equation (11) above yields a linear equation

12 $$PX=Q$$

where,

$$\begin{array}{rcl}P & = & [\begin{array}{ccc}N & \sum _{n=1}^{N}\cos \,{\delta }_{n} & \sum _{n=1}^{N}\sin \,{\delta }_{n}\\ \sum _{n=1}^{N}\cos \,{\delta }_{n} & \sum _{n=1}^{N}{\cos }^{2}{\delta }_{n} & \sum _{n=1}^{N}\cos \,{\delta }_{n}\,\sin \,{\delta }_{n}\\ \sum _{n=1}^{N}\sin \,{\delta }_{n} & \sum _{n=1}^{N}\sin \,{\delta }_{n}\,\cos \,{\delta }_{n} & \sum _{n=1}^{N}{\sin }^{2}{\delta }_{n}\end{array}]\\ Q & = & {[\begin{array}{ccc}\sum _{n=1}^{N}{{\tilde{I}}^{e}}_{nx}(x,y) & \sum _{n=1}^{N}{{\tilde{I}}^{e}}_{nx}(x,y)\cos {\delta }_{n} & \sum _{n=1}^{N}{{\tilde{I}}^{e}}_{nx}(x,y)\sin {\delta }_{n}\end{array}]}^{T}\\ X & = & {[\begin{array}{ccc}\mathop{A}\limits^{ \sim }(x,y) & V(x,y) & S(x,y)\end{array}]}^{T}\end{array}$$

in which, *δ*_*n*_(x, y) is expressed as *δ*_*n*_ for simplicity. The superscript “T” means a transpose operation. We can find out unknown values V(x, y) and S(x, y) by solving above equations and the wrapped phase map can be retrieved by

13 $${{\rm{\Phi }}}_{wx}(x,y)={\tan }^{-1}(-\frac{V(x,y)}{S(x,y)})$$

Similarly, we can also find the wrapped phase map in y-direction with modified SCPS method. Then, these wrapped phases are also unwrapped by phase unwrapping algorithm. With retrieved phases in x- and y- direction, we can find the mapping between screen and camera easily.

In case of closed fringe pattern, we could apply coordinate transform^26^ to convert this fringe into an open one. Here we only consider a closed loop fringe pattern without considering a mixed pattern of open and closed fringe. Coordinate transform of this mixed fringe pattern is a completely different problem and requires new phase retrieval strategy, so it would be out of scope of this work. With the support of coordinate transform we can convert the closed fringe into the open one. Then, we apply our modified SCPS method described above to get the wrapped phase from this open fringe. Note that we do not convert this wrapped phase from polar coordinate to Cartesian coordinate directly. Because conversion error may cause wrapped phase error and it could lead to unwrapping error. Therefore, we unwrap the phase of open fringe in polar coordinate and convert this unwrapping phase into Cartesian coordinate later. This will minimize the phase error that could happen due to Polar-Cartesian conversion.

## Results

**Figure 6:**
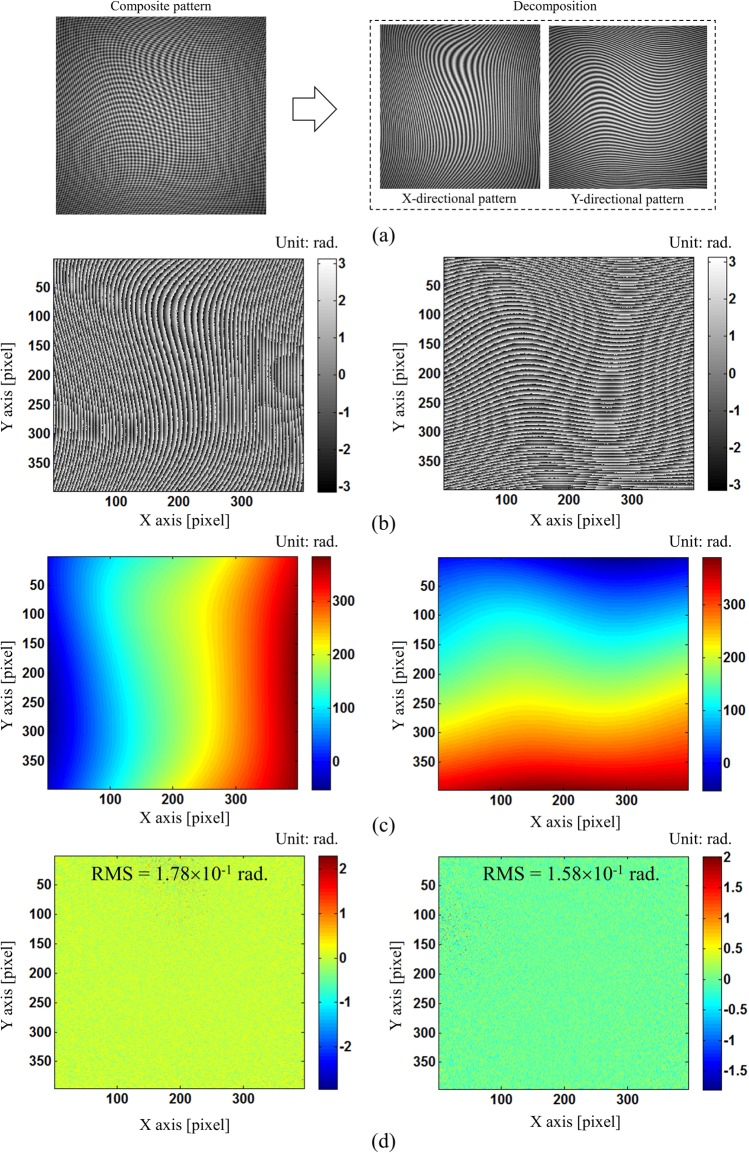
Phase retrieval from simulated composite pattern: (**a**) simulated composite pattern with Gaussian white noise level of 20 dB SNR and two single X- and Y- directional patterns after decomposing using FT method, (**b**) retrieval wrapped phase map from X- and Y- directional fringe patterns using our modified SCPS method, (**c**) unwrapped phase map from X- and Y- directional fringe patterns, and (**d**) corresponding phase difference (or error) between (**c**) and true value.

A simulation followed by experiments has been conducted to verify the correctness of our proposed method. Since our main contribution is proposing single composite pattern and modified SCPS method for phase retrieval, we generated a composite pattern which is expressed by:

14 $$I(x,y)=A(x,y)+B(x,y)\cos ({k}_{x}x+{k}_{y}y+\phi (x,y))$$

where, $A(x,y)=130\exp (-0.4({x}^{2}+{y}^{2}))$, $B(x,y)=120\exp (-0.6({x}^{2}+{y}^{2}))$ are variations in background and modulation of the fringe pattern, carrier frequencies of k_x_ and k_y_ are 1.24 rad/pixel, and $\phi (x,y)=15{\rm{peaks}}\,(x,y)$. Additionally, Gaussian white noise with the signal-to-noise (SNR) of 20 dB is added by *awgn* function using *“measured”* option in Matlab. We choose this noise level which is similar in real experiment to demonstrate the feasibility of our method. Then, by applying our proposed method, we can retrieve the phase maps in X- and Y- directions successfully without noticeable edge errors as shown in Fig. 6. And, the phase residual errors are 1.78 × 10^−1^ rad. (RMS) and 1.58 × 10^−1^ rad. (RMS) for X and Y fringe patterns, respectively. These phase residual errors are almost similar to well-known SCPS methods in the literature^23^. By increasing the carrier frequency and reducing the noise in the fringe pattern, we can obtain even higher phase accuracy. This result demonstrates that our proposed method can be applied to more complex fringe patterns in real experiment.

In our deflectometry system, we use a LCD screen with 1024 × 768 pixels resolution and its pixel size is 0.24 mm to project patterns. The images were captured by a monochromatic 8-bit digital CCD camera with 2448 × 2048 pixels resolution. Our proposed method was evaluated by performing three different experiments. In the first experiment, we measured a concave mirror with our proposed method and compared the measurement result with phase-shifting method. In order to make a fair comparison, the errors come from screen and camera non-linearity response in phase-shifting deflectometry are compensated by combining pre-distorted pattern and advanced iterative algorithm^27^. In addition, the measurement conditions are kept same. It means that we performed both measurements using the same hardware in similar environmental conditions (both measurements are performed on anti-vibration table). Therefore, we can focus on performance comparisons between two measurement algorithms without considering the effect of calibration errors. Complex and extensive calibrations of system are required if we need to obtain the absolute height of test mirror. In this experiment, we only want to compare the algorithm performance between two methods, not to measure the absolute height. Therefore, we do not put much effort on calibration steps. Detail calibrations of deflectometry system can be found in some previous papers^1,2^. Here, we can evaluate the accuracy of our proposed method by comparing with phase-shifting deflectometry, of which performance has been already verified in the previous paper^27^. After verifying the accuracy of our proposed method, we checked the system performance under harsh condition with vibrations in the second experiment. Also, we tested the dynamic performance by measuring the surface height variations of a deformable mirror in the third experiment.

**Figure 7:**
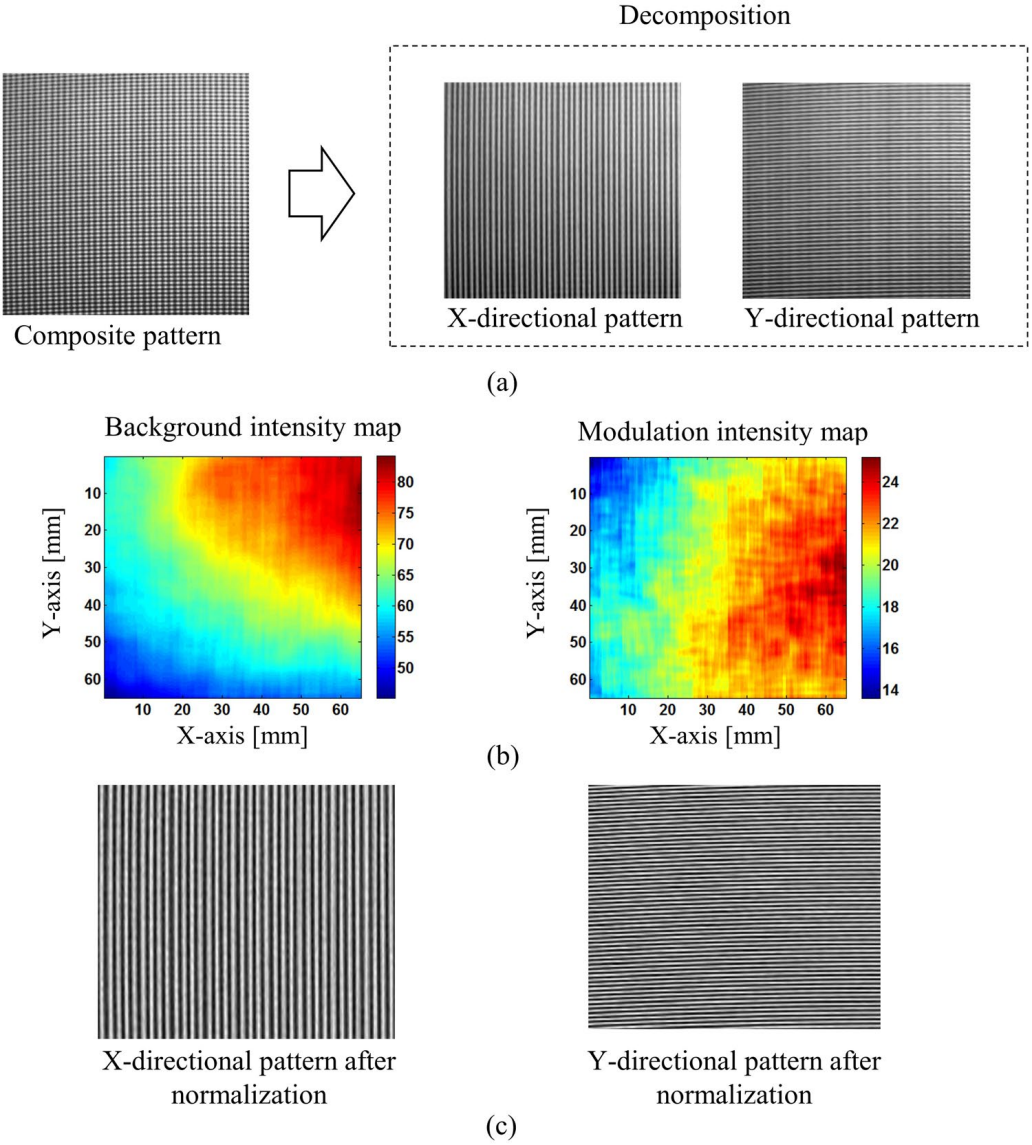
Decomposing and normalizing captured composite pattern: (**a**) captured composite pattern and x-, y-directional fringe patterns after decomposition, (**b**) background and modulation intensity variations across the measurement area after Lissajous figure and ellipse fitting, and (**c**) x- and y-directional patterns after normalization.

In the first experiment, we measured a concave mirror to verify the system performance of our proposed technique. Figure 7(a) shows the captured composite pattern and two single patterns after decomposing it. The background and modulation intensity maps obtained by using Lissajous figure and ellipse fitting clearly show the intensity variations over the measurement area. Then, the normalization process will help to make these pattern more uniform as shown in Fig. 7(c).

**Figure 8:**
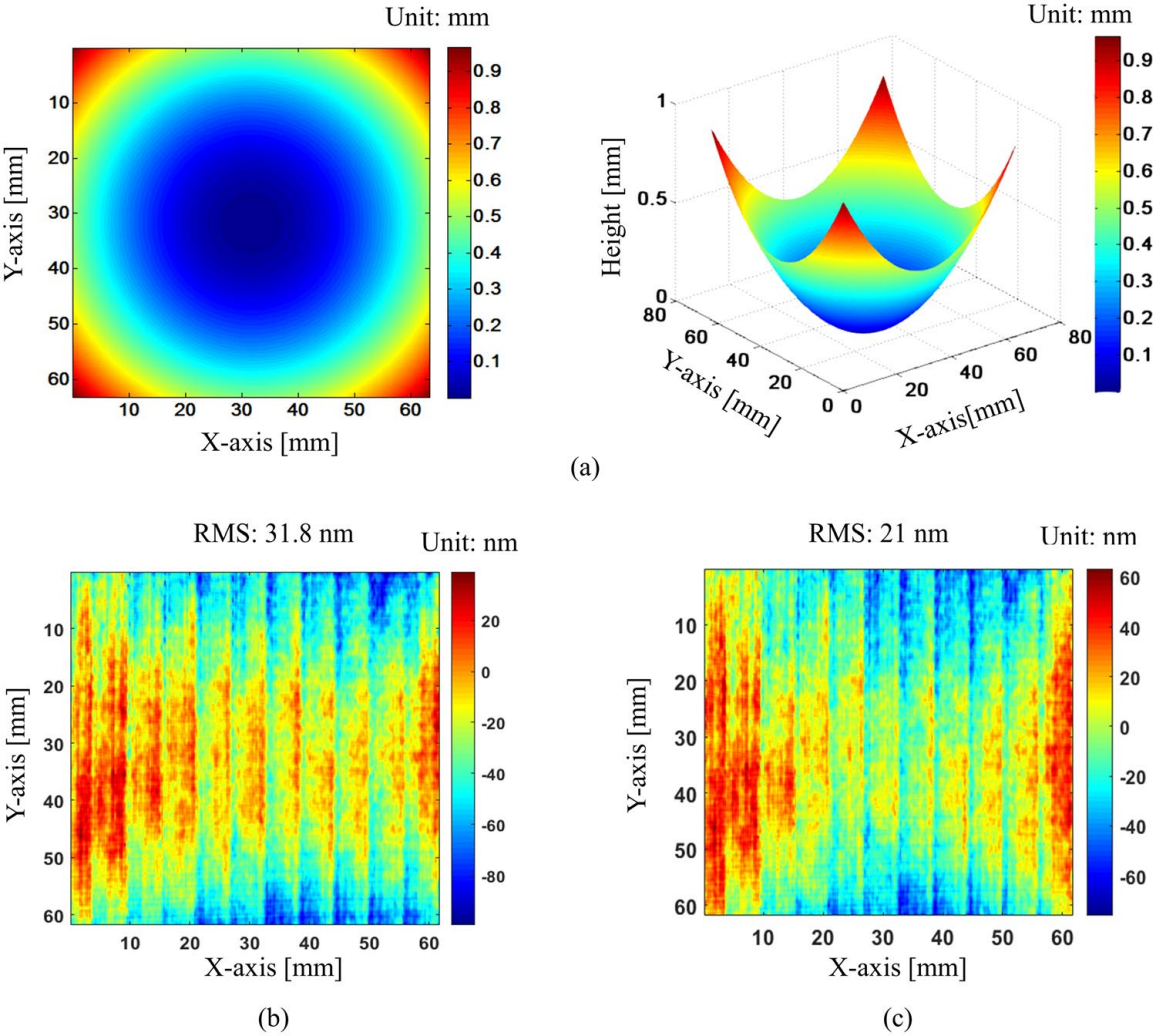
Reconstructed 3D surface profile and comparison result: (**a**) 3D surface profile of test mirror, (**b**) difference map between proposed method and phase-shifting method without normalization of fringe patterns, and (**c**) difference map between proposed method and phase-shifting method with normalization of fringe patterns.

After the x- and y-directional patterns are normalized, wrapped phases are retrieved by modified spatial-carrier phase-shifting method. Then, they are unwrapped by Goldstein unwrapping algorithm. These unwrapped phases are used to calculate the surface slopes in the x- and y-directions by the position mapping between camera and screen pixels caused by test surface. Finally, the surface shape is reconstructed from x- and y-directional slopes by modified reconstruction algorithm^28^. Figure 8 shows the reconstructed surface map and the difference between our method and phase-shifting algorithm. Comparison results show that our proposed method matches well with phase-shifting method, and the RMS value of the difference between two measurements with pattern normalization is smaller than that value without normalization (21 nm and 31.8 nm). It indicates the benefit of normalization process compensating the intensity variations of background and modulation. With severe variation, it is much more important to perform the normalization step. Our proposed method not only gives a good match but also required at least six times less than phase-shifting deflectometry in data acquisition time. It is because general phase-shifting methods require at least six images for post-processing. This is also one of the major advantages of our method.

**Figure 9:**
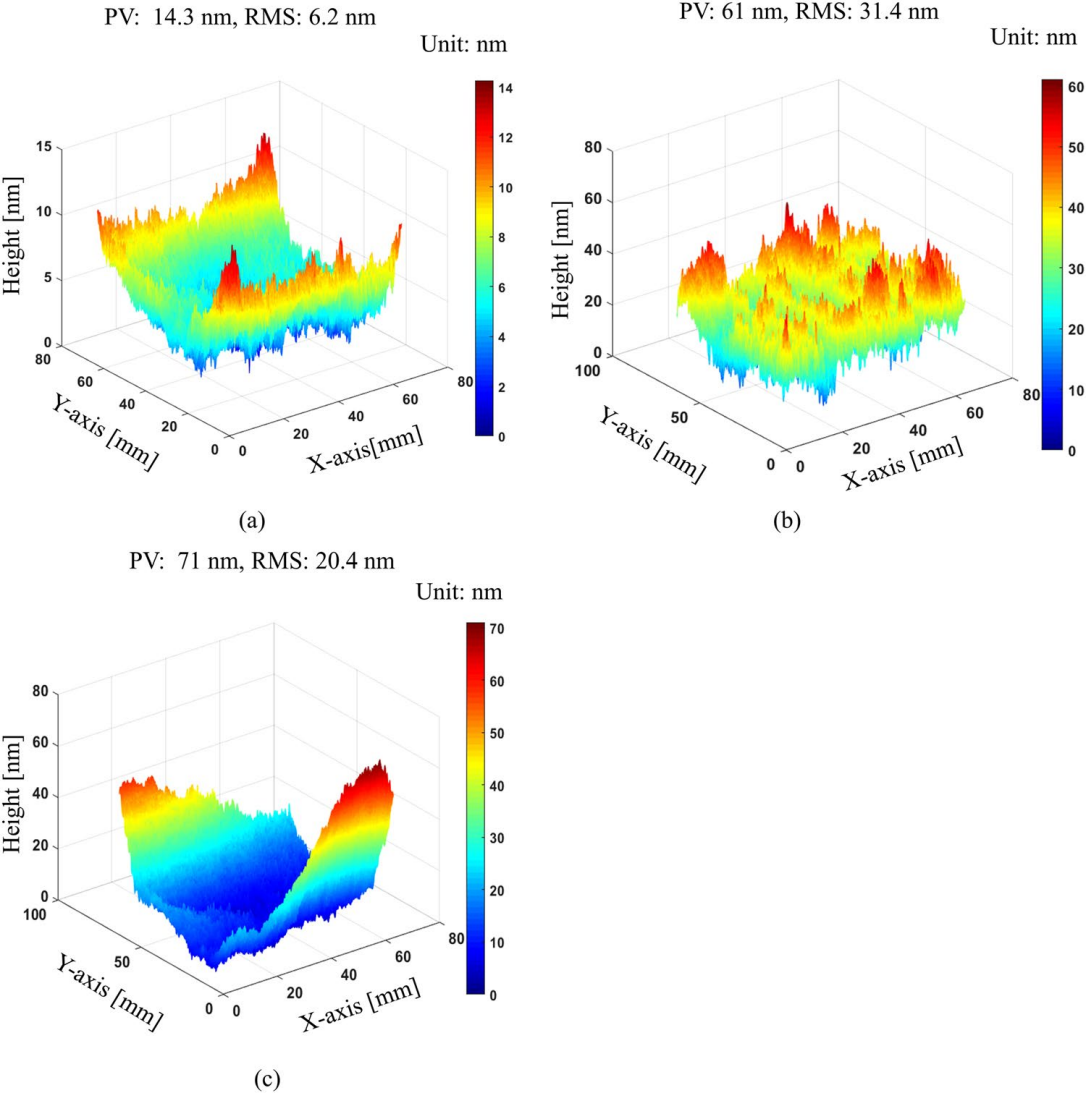
Repeatability test according to three different measurement conditions: (**a**) standard deviation map of our proposed one-shot deflectometry with anti-vibration table on, (**b**) standard deviation map of phase-shifting deflectometry with anti-vibration table on, and (**c**) standard deviation map of our proposed one-shot deflectometry with introduced vibration (anti-vibration table off).

In the second experiment, we demonstrate the robustness to environmental noise of our proposed method by measuring the mirror surface under harsh environmental conditions such as air turbulence and vibration. We first measure this mirror ten times under stable condition using anti-vibration table to evaluate its repeatability. For comparison, we also measure the same mirror ten times using phase-shifting algorithm. Then, we measure the mirror ten times under severe vibration conditions. Vibration is introduced by putting a vibrator under mirror or simply knocking on measurement table several times. Remind that the main purpose of this experiment is qualitatively to evaluate the system performance of our proposed method in harsh environmental conditions. Therefore, we introduced vibration into experiment without quantitative measurement. The Supplementary Video S1 shows the fluctuations of fringe patterns due to vibration on the test surface. Figure 9 shows the standard deviation map of ten measurement results according to different measurement conditions. Without vibration, our proposed method offers very good repeatability with PV and RMS values of 14.3 nm and 6.2 nm. Comparing with phase-shifting method, which these values are 61 nm and 31.4 nm, we can clearly see the advantage of single-shot deflectometry. With the effect of vibration, our method gives the PV and RMS values of 71 nm and 20.4 nm. These values are higher than without vibration as expected but it shows an equivalent repeatability to phase-shifting method without vibration. This result demonstrated that our proposed single-shot technique can measure vibrated surface with high accuracy.

**Figure 10:**
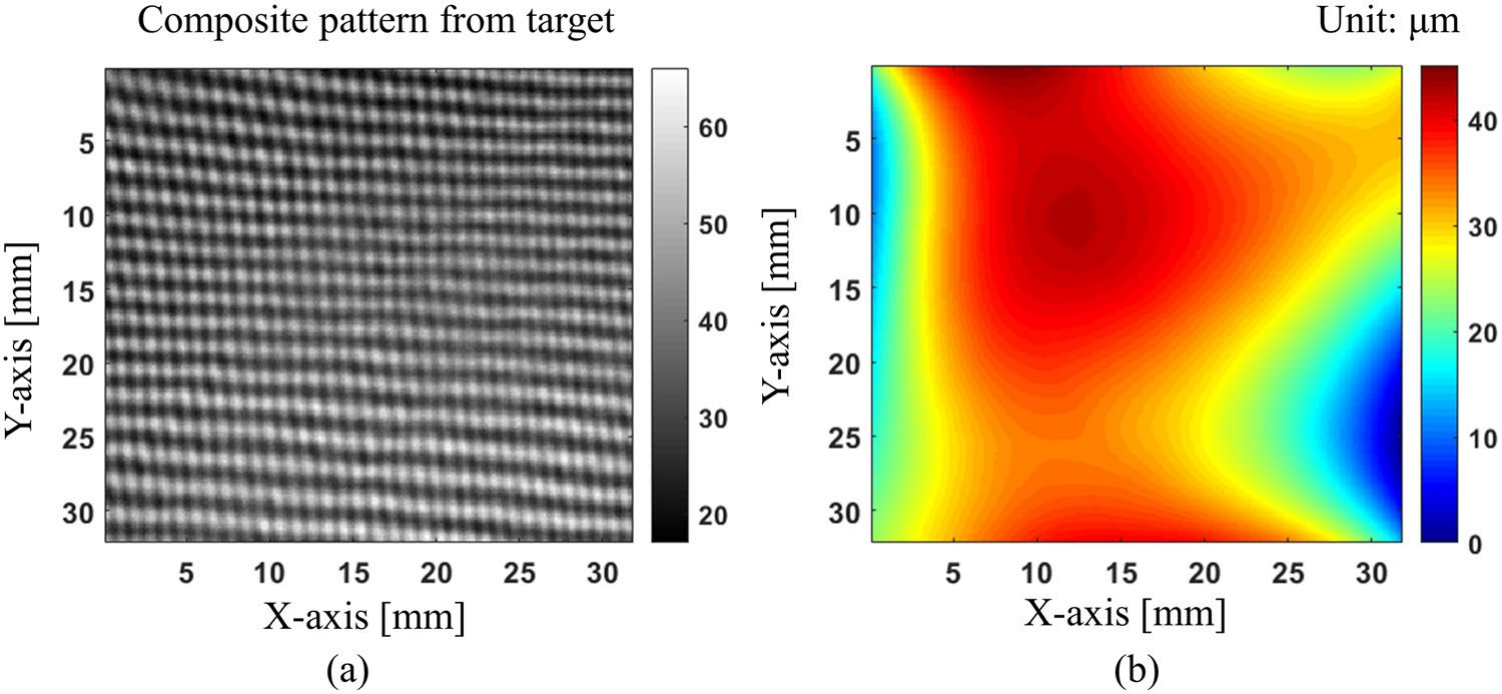
Measurement results of deformable mirror: (**a**) Distorted pattern reflected by deformable mirror and (**b**) reconstructed surface of deformable mirror.

The last experiment demonstrates that with the proposed technique, it is possible to measure the surface deformation in real time while it is very difficult or even impossible with phase shifting method. In this experiment, we measured the deformable mirror by changing its surface profile continuously. The surface profile is deformed according to the change of the load applied to the mirror. We captured 34 continuous images of the deformed shape of the mirror. Note that our main purpose is qualitatively measuring surface deformation using our proposed method. We need to confirm that our method could detect small surface deformation when changing the load applied to the deformable mirror in real time. Therefore, we do not focus too much on the absolute surface height deformation. Figure 10 shows the distorted composite pattern on the deformable mirror captured by camera and its corresponding reconstructed surface map.

**Figure 11:**
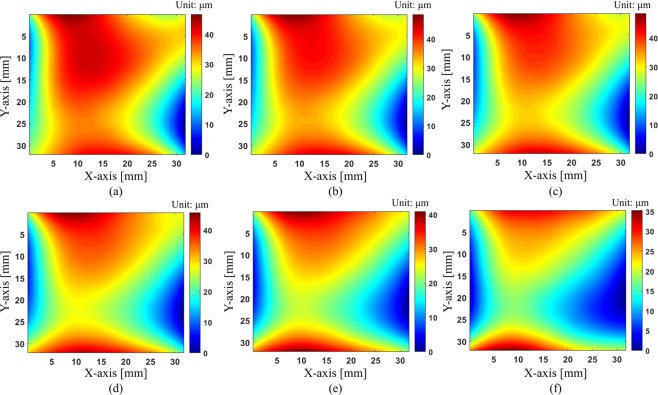
Reconstructed surface maps of the deformed shape of the mirror measured by our proposed one-shot deflectometry as time elapses: (**a**) 0.15 s, (**b**) 0.3 s, (**c**) 0.45 s, (**d**) 0.6 s, (**e**) 0.75 s, and (**f**) 0.9 s.

The Supplementary Video S2 demonstrates how the distorted pattern looks like when the surface profile changes on the left. By processing these continuous distorted images, we can reconstruct the movies of surface deformation on the right. Figure 11 shows the reconstructed surface maps of the deformation of mirror according to time elapse. The peak-to-valley value of surface height in this experiment is less than 50 μm and our technique can monitor a small change of surface profile. Because we need only a single image for data processing, the measurement speed of our method depends on the frame rate of a camera. Therefore, we can increase the measurement speed by using a higher frame rate camera.

## Discussion

**Figure 12:**
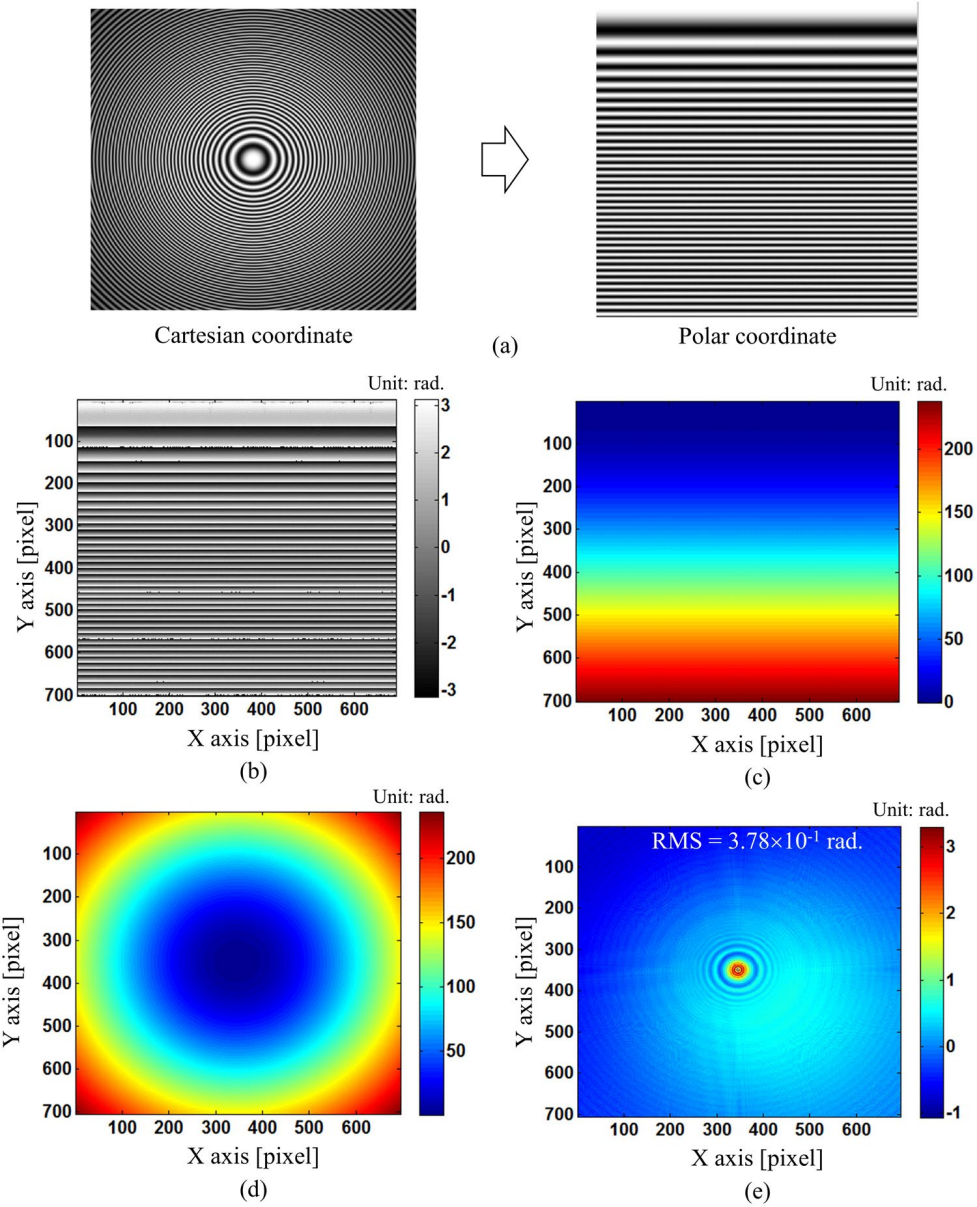
Phase retrieval from closed loop fringe pattern using our modified SCPS method: (**a**) closed loop fringe pattern and open fringe pattern transformed by using Cartesian-Polar coordinate transform, (**b**) retrieved wrapped phase of open fringe pattern in polar coordinate, (**c**) unwrapped phase of (**b**) in polar coordinate, (**d**) unwrapped phase after converting (**c**) to Cartesian coordinate, and (**e**) phase difference (or error) between (**d**) and true value.

Although our proposed method can work well with open fringe pattern, it is unclear about suitability of our technique on closed loop fringe pattern which might happen in real measurement. Since it is very difficult to get the closed loop fringe pattern with high density in deflectometry by experiment, we verified our technique by simulating a closed loop fringe pattern, which is expressed by

15 $${I}_{sim}(x,y)=a(x,y)+b(x,y)\cos (2\pi n\exp [({x}^{2}+{y}^{2})])$$

where $a(x,y)=130\exp [-0.4({x}^{2}+{y}^{2})]$, $b(x,y)=120\exp [-0.6({x}^{2}+{y}^{2})]$ are variations in background and modulation of the fringe pattern; x, y coordinates are from −1 to 1, n = 60 is the number of fringes. We straighten out this closed pattern by applying coordinate transform and then retrieve the wrapped phase using our modified SCPS method. This wrapped phase is unwrapped in Polar coordinate before transforming back to Cartesian coordinate. Figure 12 shows the process of obtaining the phase from closed loop fringe pattern using our modified SCPS method. Our method can successfully retrieve the phase from this closed pattern with the phase error of 3.78 × 10^−1^ rad RMS. Most of error comes from the center of closed loop fringe pattern since the fringe density in this region is not high enough to apply our modified SCPS method. As shown in Fig. 12(a), the spatial frequency of open fringe in the upper part corresponding to the center of closed fringe is not high compared to the lower part corresponding to the outer region of closed fringe.

If closed loop fringe pattern appears in real measurement including in dynamics, it is impossible to decompose the composite pattern into x- and y-directional patterns using Fourier-based technique because of overlapping problems in frequency domain. In this case, instead of projecting composite pattern, we should project two separate sinusoidal fringe patterns in x- and y-direction at high speed. Then, if captured fringe patterns have closed loop, we can apply coordinate transform to make it opened and retrieve the phase using our modified SCPS method. It will take a little bit more time than just using one composite pattern, but it is still possible for high speed measurement and even real time since we need two shots only.

Our main goal here is presenting the feasibility of new composite pattern in single-shot deflectometry with gray scale camera. Therefore, we do not put much effort on calibration as mentioned in the experimental section. In order to achieve higher accuracy, we need a very carefully calibration process which takes into account the shape of screen profile, camera’s distortion and imaging aberrations. Moreover, the geometrical parameters are also needed to be measured accurately. Systematic errors could be removed by using flat reference surface. It means that, by measuring the flat reference surface and subtract this surface variation from actual measurement, we can obtain the surface profile with higher accuracy. As shown in Fig. 8(c), without much effort on calibration, we still get the good agreement between our proposed method and phase shifting method.

There are two main types of errors in this proposed method: systematic errors or common errors to all deflectometry systems, and errors that are unique to single-shot composite pattern. Common errors include screen deformation that distorted the display fringe pattern causing systematic errors, camera and screen nonlinearity response causing print-through artifact. Our proposed composite pattern also have its own error sources. The main error source of this method comes from SCPS phase retrieval method with the support of Fourier transform which often causes edge error because of frequency leakage. However, this kind of error source is mitigated by subtraction operation as indicated by Z. Dong and H. Cheng^19^. If we do not concern about processing time, it is possible to replace this hybrid method by other spatial carrier frequency method which does not apply FT method, such as J. Xu *et al*.^20^ which uses iteration strategy to obtain the local phase shifts. Therefore, it is not suffered by edge error anymore and thus we can avoid this type of error in our method. Since the sub-fringe patterns are constructed from consecutive pixel positions, phase retrieval accuracy from our proposed method is effected by noise significantly which is similar to all SCPS methods^19–21^. Therefore, in the case of severe noise, captured fringe pattern should be denoised before performing our method to obtain more accurate phase.

In summary, our proposed method has some main advantages compared to previous ones:

1. This method is much faster than the fastest phase shifting method in deflectometry (three phase-stepping) which require at least six images. Our method only need a single image for processing.
2. This method is not affected by the color of the object contrary to some other single-shot methods using color camera to multiplexing fringe pattern.
3. Little or not suffer from edge effect due to frequency leakage contrary to other Fourier-based single-shot techniques

However, our proposed method also has its own disadvantages:

1. More sensitive to noise than instantaneous method and FT method. Therefore, we need to apply some noise removal method to the fringe pattern before performing proposed method in the case of severe noise.
2. Computation time for phase-retrieval is longer than some previous methods because of normalization process and least-squares iteration.
3. The accuracy will reduce if closed loop fringe pattern appears and we need to take two shots instead of single shot due to the theoretical limitation of Fourier transform when decomposing the composite pattern including closed patterns.

## Conclusion

We have presented an innovative single-shot deflectometry technique and verified its performance by comparing various experimental results with phase-shifting method. Our proposed method not only has equivalent performance with phase-shifting method but can also measure surface even under harsh environmental conditions in real-time. We discussed various error sources which are common for all deflectometry system and specific to one-shot deflectometry only. We also pointed out the main advantages and disadvantages of our proposed method compare to previous techniques. It is anticipated that our proposed technique will be widely used as in-line industrial metrology tool capable of measuring complex surfaces including free-form surfaces.

## Supplementary information

the fluctuations of fringe patterns due to vibration on the test surface.

3D surface profile measurement of dynamic object
